# Money a Medicine

**Published:** 1855-06

**Authors:** 


### MONEY A MEDICINE.

Prosperity is the best pill, it wakes up the failing pulses of life, and renovates the whole machinery of man. Take two poor men who are equally ill, to whom exercise is alike applicable, condemn one to the unendurable drudgery of walking a mile thrice daily to a certain post, and when he gets there to turn round and walk back again; and let another spend an equal time in collecting bills, or obtaining subscriptions at a per centage, which clears him ten dollars a day, if he is diligent; it is easy to conjecture which of the two will convalesce the more rapidly. One thing I am certain of, making money helps *me* amazingly, it is the elixir of mind and body both. This idea of the hygienic value of money on men is strikingly illustrated in the report of M. Vellerme, as the Secretary of the Poor Law Commissioners in Havre, where the average age of the rich is twelve years greater than that of the poor.

| | | | | | |
|---|---|---|---|---|---|
| 1088 | prosperous persons died at an average age of | | | 42 | years. |
| 4791 | middling class | " | " | 29 | " |
| 19849 | poor | " | " | 20 | " |

Therefore, as it is easier to take money than to take pills, I advise my readers, one and all, as a means of long life, to get rich by prudent industry and honorable economy.

